# The Impact of Behavioral Economics-Based Counseling and Mobile Phone Text Educational and Reminder Messages on the Use of Modern Family Planning in Jordan: A Cluster Randomized Controlled Trial

**DOI:** 10.3390/healthcare11091314

**Published:** 2023-05-03

**Authors:** Heath Prince, Yousef S. Khader, Yara A. Halasa-Rappel, Sara Abu Khudair, Mohammad Alyahya, Nihaya Al-Sheyab, Khulood K. Shattnawi, Rana AlHamawi, Kelley Ready

**Affiliations:** 1 Ray Marshall Center for the Study of Human Resources, Lyndon B. Johnson School of Public Affairs, The University of Texas at Austin, Lake Austin Blvd., Austin, TX 78712, USA; 2 Department of Community Medicine, Public Health and Family Medicine, Faculty of Medicine, Jordan University of Science and Technology, Irbid 22110, Jordan; 3 ForHealth Consulting, UMass Chan Medical School, Shrewsbury, MA 01545, USA; 4 Eastern Mediterranean Public Health Network, Amman 11196, Jordan; 5 Department of Health Management and Policy, Faculty of Medicine, Jordan University of Science and Technology, Irbid 22110, Jordan; 6 Allied Medical Sciences Department, Faculty of Applied Medical Sciences, Jordan University of Science and Technology, Irbid 22110, Jordan; 7 Maternal & Child Health Nursing Department, Faculty of Nursing, Jordan University of Science and Technology, Irbid 22110, Jordan

**Keywords:** behavioral economics, family planning, modern family planning methods, text-based intervention

## Abstract

Background: Favorable attitudes toward modern family planning methods (MFPMs) among Jordanian and Syrian women do not always translate into behavioral changes, and the availability and cost of MFPMs do not appear to be related to either prior stalls in fertility rates in Jordan or to the current and likely temporary decline in fertility rates. This study aimed to determine whether behavioral economics (BE)-based family planning interventions influence the use of any family planning method, MFPMs use, continuation of MFPMs use, and pregnancy rates among women in Jordan. The BE-based family planning interventions included personalized text messaging and augmented counseling based on framing and identity-priming BE principles, with their effects tested over a 9-month period in the postpartum period following the birth of a child. Methods: A parallel-group cluster randomized controlled trial was conducted to compare two interventions, augmented counseling based on framing and identity-priming BE principles and personalized mobile phone text messages reminders, aiming to improve the utilization of MFPMs among postpartum women over status quo family planning services in comprehensive health centers in the north of Jordan. Results: In total, 1032 participated in the study: 295 women in the control group; 326 women in Intervention Group 1, which received only augmented counseling; and 411 women in intervention Group 2, which received augmented counseling and monthly text messages. The rates of using MFPMs in the counseling group and the counseling and messages group 3 months (54.7% and 57.1%, respectively), 6 months (50.0% and 51.7%, respectively), and 9 months (49.5% and 52.0%, respectively) were significantly higher than the rates among women in the control group (40.6% at 3 months, 37.6% at 6 months, and 34.3% at 9 months). Overall, 26.8% of women in the control group, 42.1% of women in the counseling-only group, and 45.2% of women in the counseling and messages group used MFPMs continuously for all 9 months. At 9 months, the pregnancy rate was significantly much higher in the control group (13.7%) compared to women in the counseling-only group (7.0%) and to women in the counseling and messages group (7.4%). Conclusions: Simple BE-based interventions can be effective methods for enhancing the use of MFPMs and maintaining the anticipated decline in Jordan’s total fertility rate.

## 1. Introduction

Utilization patterns of modern family planning methods (MFPMs) are crucial in evaluating the overall coverage and success of family planning programs and services. Jordan has achieved great success in improving access to, provision of, and use of family planning methods and services [1], particularly between 1990 and 2012, when the contraceptive prevalence rate (CPR) increased from 40% to 61%, with the modern CPR increasing from 27% to 42% and the traditional CPR increasing from 13% to 19% [2]. Following this increase in CPR, the total fertility rate (TFR)—inversely related with CPR [3]—declined from an average of 5.6 children per woman in 1990 to 3.5 in 2012 but with a long fertility stall between 2002 and 2012 [2].

The most recent data from the 2017–18 Jordan Population and Family Health Survey (JPFHS) showed a decline in TFR to an average of 2.7 and an unusual decline in CPR to 52% [2]. Moreover, the desired average family size, which is often considered an indicator of fertility levels [4,5], is still high at 4 children for both women and men in Jordan [2]. The decline in contraceptive use in Jordan could be attributed to a combination of factors; these include several barriers to MFPMs use cited by women, such as compliance with social and cultural norms regarding childbearing and prioritization of male children, inconsistency of the counseling process, experienced and anticipated adverse side effects of using MFPMs, and family pressures from husbands and in-laws [6,7]. Other indirect factors include Jordan’s consistently high educational achievement rates for both women and men as well as the current tendencies for Jordanian women to marry relatively later in life [4]. However, the role of cost is potentially less important, as family planning services are provided free of charge to Jordanians and at a subsidized rate to non-Jordanians by the Ministry of Health.

Studies and national reports therefore reported the need for interventions to increase the use of MFPMs to achieve the 2030 target of 2.1 TFR set by Jordan’s Higher Population Council [4,5,8]. An assessment of Jordan’s family planning services, based on a desk review and key informant interviews, revealed the need for more client-centered family planning models, behavior change campaigns, and programmatic activities that address social norms and MFPMs misconceptions and biases [9]. One of the interventions that has proven effective in supporting the adoption of healthy family planning practices is social and behavioral change (SBC), which articulates a deep understanding of human behavior and uses theory and practice from a variety of fields, including behavioral economics (BE), communications, social psychology, anthropology, sociology, human-centered design, and social marketing [10]. The complex nature of family planning behaviors, over which the confluence of culture, policies, knowledge, and other factors exerts enormous influence, makes the mechanism by which positive intentions are translated into actual behavioral change regarding family planning difficult to understand unless the beliefs that undergird seemingly irrational decisions are first fully understood [11]. BE appears to be a promising strategy for strengthening family planning programs and improving decision making, as it uses insights from psychology and economics to explain human behavior. BE recognizes that people make irrational decisions that are often predictable and offers several approaches to counteract the triggers for these irrational behaviors [11,12]. In addition, BE can help explain why some individuals or couples may choose not to use family planning methods, despite the potential benefits of doing so. Some possible explanations based on behavioral economics principles include present bias. For some individuals, the perceived benefits of having children right now may outweigh the potential benefits of delaying or spacing out pregnancies. This may be particularly true in situations where cultural or social norms emphasize the importance of having children early in life. Another explanation is discounting future consequences. Some individuals or couples may discount the future benefits of using contraception (such as improved health outcomes, increased economic opportunities, and better educational outcomes for their children) in favor of the immediate benefits of having children.

Here, the anticipated effect of BE strategies is to strengthen the link between women’s family planning intentions and actual behavioral outcomes [11]. BE has been used in a variety of health contexts, including promoting healthy eating, weight control, and physical activity [13] and tobacco control [14], and has also been further applied in the realm of family planning methods and reproductive health [15]. Enhancing women’s use of MFPMs is acknowledged as a need in Jordan, and increasing contraceptive use is arguably of demonstrable benefit to developing and low- and middle-income countries from both health and economic perspectives [16,17].

However, the challenge for researchers is to design interventions that adhere to the Cairo consensus of improving women’s reproductive health and sexual rights and the rights of the vulnerable youth and refugee populations while piloting methods for increasing the number of women who effectively use MFPMs [18]. Thus, this study employed a culturally sensitive, inclusive, and anthropologically based approach that incorporated principles from the BE field.

There is a growing interest in using BE interventions for behavior change in health, but empirical evidence is lacking, particularly in the context of family planning [11]. Thus, this study aimed to determine whether BE-based family planning interventions influence the use of any family planning method, MFPMs use, continuation of MFPMs use, and pregnancy rates among women in Jordan. The BE-based family planning interventions included personalized text messaging and augmented counseling based on the BE principles of *framing* and *identity priming*, with their effects tested over the 9-month period postpartum period following the birth of a child. 

## 2. Materials and Methods

### 2.1. Study Setting and Population

This study was conducted in the Irbid and Mafraq governorates, which are located in the northern region of Jordan. According to the Jordanian Department of Statistics, the estimated 2017 population of Irbid was 1,867,000, while the estimated population of Mafraq was 580,000 [19]. The selection of the two governorates was based on criteria related to the adequacy of representation of women belonging to the lower two income quintiles [2,20] and being among the regions with the highest density of Syrian refugees living within the host communities in urban, peri-urban, and rural areas [21].

### 2.2. Study Design

A parallel-group cluster randomized controlled trial was conducted to compare two interventions, “Intervention 1” and “Intervention 2”, aiming to improve the utilization of MFPMs among postpartum women in comprehensive health centers in the north of Jordan with a control group with status quo family planning services. This design avoided contamination that could have arisen from within-cluster randomization of women, clients, or providers. The primary sampling unit was comprehensive health centers. The secondary sampling unit included postpartum women who attended child health/immunization visits during the period April–September 2018, with the timing of delivery being within 6 weeks or less. According to the World Health Organization (WHO), women in the postpartum period show higher proportions of unmet needs for family planning. Moreover, the postpartum period provides a unique opportunity to address women’s contraceptive needs and provide high-quality information on contraceptive options while women are engaged in care.

The sample frame was composed of a list of health centers in the selected districts, obtained from the Directorate of Primary Health Care at the Ministry of Health. Six districts (comprising the sampling strata) in Irbid governorates have a total of 11 centers. Three districts in the Mafraq governorate have a total of 20 centers. From each district, 1 center was selected using a simple random sampling method for a total of 9 comprehensive health centers, including 3 centers used as controls. The selected centers were randomly allocated among the two intervention and control arms.

Women were enrolled in the study consecutively during their visits to comprehensive health centers (within ≤6 weeks of delivery) either for postpartum care or for their child’s routine immunization service. Women were included in the study if they met the following inclusion criteria: (1) postpartum married women at reproductive age, (2) resided in the selected two governorates, (3) visited the comprehensive health centers for their child’s (6 weeks of age or younger) routine immunization services, (4) owned a mobile phone, and (5) agreed to give consent to participate in the study. The vast majority of women reported not using contraceptives during their first visit. The few women who reported using contraceptives by this time were excluded.

### 2.3. Sample Size

Based on the 2012 JPFHS, 42% of Jordanian married women used MFPMs [20]. To detect an increase of 10% in use of MFPMs in the intervention groups at a significance level of 5% and with 80% power, a sample size of at least 305 women in each arm was needed. The number of women selected from each center was proportional to the number of women attending the health centers. 

### 2.4. Intervention

Our intervention was composed of two main components. First, and to address the shortcomings in counseling, we designed a counseling protocol for midwives and provided training in its use that augmented existing counseling methods by including the most current information on traditional family planning methods and MFPMs including long-acting reversible contraceptives, short-acting methods, and permanent methods. In addition, the protocol included language reflecting two approaches from the BE field: *framing* and *identity priming*. *Framing* assumes that how information is conveyed and presented is critical to how people make decisions, and it assists in countering misperception of risks by highlighting the appeal of some outcomes over others [15,22]. 

*Identity priming* is an approach that appeals to gender, race, or role in order to make certain outcomes more appealing by assuming that actions are often influenced by unconscious and positive cues that can stimulate a person’s decisions [15,22]. In the context of increasing women’s use of MFPMs, our augmented counseling protocol included appeals to women as responsible mothers, rather than simply wives, and to men as fathers, rather than simply husbands, who naturally want to provide the best possible quality of life for their children.

The second component of the intervention also employs a BE approach: *reminders*. Text message reminders have been shown to be effective in various health care contexts, including the use of contraceptives [23]. Reminder messages are a useful tool for implementing nudges, which is an application of insights of BE to subtly influence decisions without restricting individual freedom of choice [24]. Reminder messages can help people take intended actions by overcoming obstacles that prevent them from taking a particular action, including those related to competing commitments, procrastination, inertia, and forgetfulness [24]. The text messages were sent monthly in the local language (Arabic), reminding women of the benefits of MFPMs and encouraging them to follow up with midwives to address any questions. The messages covered essential topics related to MFPMs such as types of MFPMs; their benefits to women, children, and the family; how to use them; and their contraindications. Some messages were used to deal with myths and misconceptions related to MFPMs. 

Intervention Group 1 received augmented counseling only, while Intervention Group 2 received both augmented counseling and monthly text messages, with the aim of isolating the marginal effects of the text messaging intervention. Women in the control arm received routine counseling. Women in the two intervention groups and in the control group received follow-up phone calls from project staff at 3, 6, and 9 months. Each of the two intervention groups was compared to each other and to a control group of women who received services at clinics per the status quo.

### 2.5. Data Collection

#### 2.5.1. Baseline Data Collection

The questionnaire was developed in Arabic based on a review of previous research studies and structured into different modules. A group of researchers, clinicians, and laypeople reviewed and commented on the final version of the questionnaire to ensure face and construct validity. To test the instrument and the procedure, a pilot study was conducted on 30 women in each group. Based on feedback from the women, the questionnaire was rephrased and modified. 

With the approval of health center administrators, two midwives from each selected health center were extensively trained on the study procedures and on completing the baseline questionnaire. Midwives verified the inclusion criteria, explained the study to participants, obtained written consent, and completed the baseline questionnaire with the participants before counseling. Data collection was monitored regularly by the study investigators. On the other hand, a trained person was recruited to conduct all follow-up calls. The baseline questionnaire included questions on a mother’s identity, her socioeconomic and clinical characteristics, her newborn (including gender, birth weight, and gestational age), her awareness/knowledge of MFPMs, her attitude towards and past experience with contraceptives, her husband’s support, and her intention to use contraceptives going forward. The baseline data were collected during the period April–September 2018. During the period of data collection, researchers met periodically with interviewers and respondents to review their experience.

#### 2.5.2. Follow-Up Data Collection

At months 3, 6, and 9 postbaseline, a trained person conducted individual interviews over the phone with participants to collect information about the main study outcomes and intervention satisfaction. The interviewer asked women in the intervention groups to evaluate their experience of the intervention and to evaluate the intervention they received, including the counseling and text-messaging components. At 3 months postbaseline, women were asked about their awareness of contraceptives and traditional family planning methods, their attitude towards contraception, their partner’s support in using MFPMs, and the accessibility of family planning services. At months 3, 6, and 9 postbaseline, women were asked about their current use of family planning methods, the type of methods used, current practices of child feeding, how long they intend to continue using contraceptives, whether or not they changed the method of contraception in the last 3 months, and the place from which they receive their chosen family planning method. Women who did not report the use of family planning methods were asked about the reason for not using any contraception and about their intention to use and the methods they intend to use. A single questionnaire was used for data collection at baseline and follow-up, which included a separate module on identity information to allow for follow-up.

### 2.6. Ethical Approvals

Ethical approvals were obtained from the Institutional Review Board (IRB) at the University of Texas at Austin (USA) (Protocol code J682 and date of approval 12 January 2018), the Jordan University of Science and Technology, and the Jordan Ministry of Health (approval number 4/109/2017). To ensure that they would benefit from a BE approach, participants were clearly informed about the nature of the study, protected from harm in participating, and involved in guaranteeing that the decision outcomes are those that they truly desire. Researchers ensured that the participants were not unduly burdened by the data collection. Participants were approached in a culturally sensitive manner, with particular attention paid to the gender of the interviewers. Researchers informed participants that their participation was voluntary and that they had the right to withdraw at any time. Confidentiality and anonymity of the respondents were protected through a process-oriented approach, which has three different aspects of informed consent: the provision of information by the researcher or service provider, the participant’s comprehension of the information, and the participant’s voluntary decision making. Researchers provided this information in an understandable language.

The study was not registered, because the regulations governing clinical trials in Jordan request registration of clinical trials that involve administering medications only.

### 2.7. Statistical Analysis

Statistical analysis was performed using SPSS IBM (version 20). Data were described using the mean and standard deviation (SD) for continuous variables and frequency and percentages for categorical variables. Chi-square was used to compare the baseline characteristics of mothers and outcome variables in the three study groups. Binary logistic regression was conducted to test for the difference in the use of MFPMs at different points of time after adjusting for important variables. All possible confounders were adjusted for in the multivariate analysis. A *p*-value of < 0.05 was considered statistically significant.

## 3. Results

### 3.1. Baseline Characteristics

In total, 1032 participated in the study: 295 women in the control group; 326 women in Intervention Group 1, augmented counseling only; and 411 women in Intervention Group 2, augmented counseling and monthly text messages. Table 1 shows the sociodemographic and clinical characteristics of women at the baseline. Women in the control group were slightly older than women in the other two groups; the mean ages were 28.5 years for women in the control group, 27.1 years for women in the counseling group, and 27.7 years for women in the counseling and messages group. The vast majority of women in the three groups were Jordanians, and approximately 21% were Syrian. While they differed in some respects, the women in the three groups were similar in other important characteristics such as occupation, income, the number of male and female children in the family, and other clinical characteristics. 

### 3.2. Pregnancy-Related and Obstetric Characteristics

Regarding the pregnancy-related and obstetric characteristics of women at the baseline (Table 2), women in the three groups differed significantly in the number of antenatal visits, the time at the first antenatal visit, and the perception of the timing of the last pregnancy. However, across the three groups, the women were similar in many important characteristics that might be related to contraceptive use, such as whether the birth was a single birth or the birth of multiples, the gender of the baby, the place of delivery, the type of delivery, prematurity, and low birthweight delivery. 

### 3.3. Current Use of Any Family Planning Methods

At 3 months of follow-up, the vast majority of women in the counseling group (92.6%) and in the counseling and messages group (91.3%) and three-quarters of women in the control group (75.3%) reported that they were using at least one method of family planning. The percentage of women who reported using any method at 6 and 9 months was less than that at 3 months; while approximately 85% of women in the two intervention groups were using family planning at 6 and 9 months, 70.8% and 68.6% of women in the control group were using family planning methods at 6 and 9 months, respectively. The rate of family planning use among women in the two intervention groups was significantly higher than that among women in the control group at all follow-up times. However, women in both intervention groups did not differ significantly in their use of family planning at all follow-up points. 

### 3.4. Current Use of MFPMs

**Figure 1:**
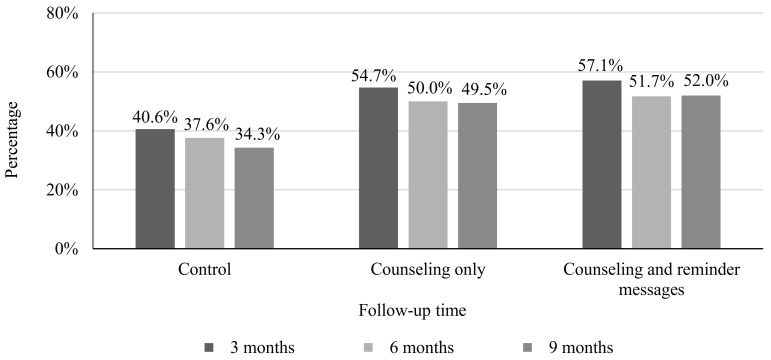
The rate of using any MFPM at 3, 6, and 9 months of follow-up in the two intervention groups and the control group.

The rates of using MFPMs in the counseling group and the counseling and message group at 3 months (54.7% and 57.1%, respectively), 6 months (50.0% and 51.7%, respectively), and 9 months (49.5% and 52.0%, respectively) were significantly higher than the rates among women in the control group (40.6% at 3 months, 37.6% at 6 months, and 34.3% at 9 months) (Figure 1). 

### 3.5. Continuation of Using Modern Family Planning Methods

**Figure 2:**
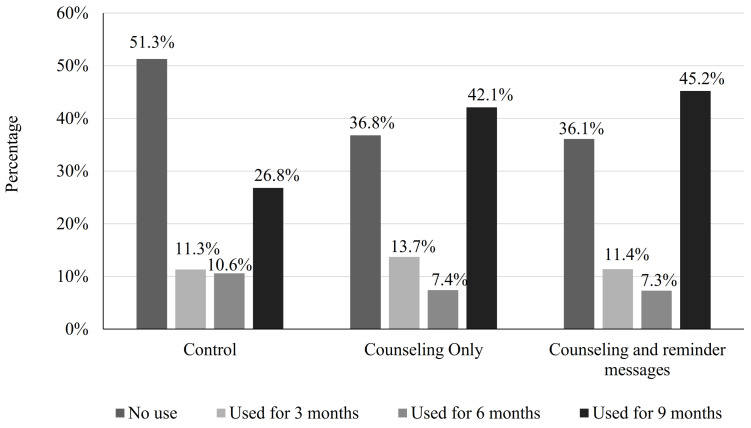
Continuation of use of MFPMs in the two intervention groups and the control group.

Almost 36.8% of women in the counseling-only group, 36.1% of women in the counseling and messages group, and 51.3% of women in the control group opted *not* to use MFPMs. Overall, 26.8% of women in the control group, 42.1% of women in the counseling-only group, and 45.2% of women in the counseling and messages group used MFPMs continuously for all 9 months (Figure 2).

### 3.6. Multivariate Analysis

In the multivariate analysis, women in the counseling group and women in the counseling and messages group were significantly more likely than women in the control group to use modern methods at 3 months, 6 months, and 9 months (Table 3). Intention to use family planning methods at the baseline was significantly associated with increased odds of using modern methods at all periods of follow-ups. Being aware of family planning methods available at the clinic, having ever used any modern contraception, having delivered at home, and having a single birth (vs. bearing twins or more) were associated with higher odds of using modern methods at 6 and 9 months only. Reason for current visit (postpartum care vs. immunization), husband’s education of high school or less, income of <JOD 400 vs. ≥JOD 400, feeling that the timing is wrong for the last pregnancy, and having heard about family planning and breastfeeding were significantly associated with increased odds of using modern methods at 3 months of follow-up.

### 3.7. Pregnancy

**Figure 3:**
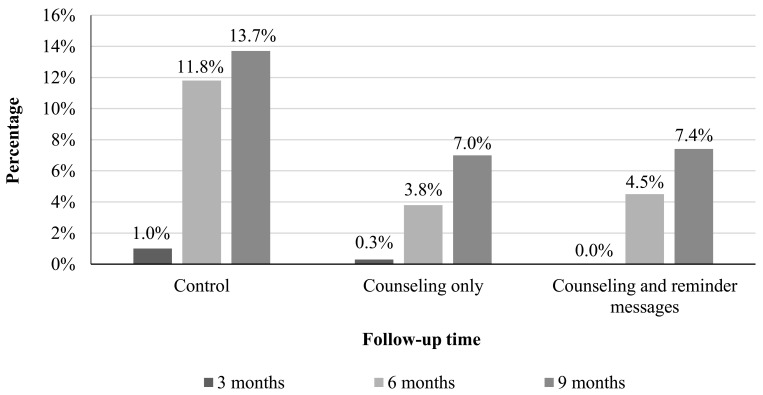
Pregnancy rates among women in the three study groups at 3, 6, and 9 months of follow-up.

Figure 3 presents the pregnancy rate among women in the three study groups at different follow-up times. At 3 months after the initial visit to the health center, 1% and 0.3% of women in the control group and in the counseling group, respectively, were pregnant. None of the women in the counseling and message group were pregnant at 3 months. At 6 months, 11.8% of women in the control group, 3.8% of women in the counseling group, and 4.5% of women in the counseling and message group were pregnant. At 9 months, the pregnancy rate was significantly much higher in the control group (13.7%) compared to women in the counseling group (7.0%) and women in the counseling and message group (7.4%).

## 4. Discussion

A BE-based family planning intervention was used to increase the use of MFPMs among women. Results showed that the rate of MFPMs use was significantly higher in the two intervention groups—those who received only the augmented counseling and those who received the augmented counseling and text messages—than in the control group with routine counseling. Also, continuation of MFPMs use was higher and pregnancy rates were lower in the two intervention groups compared to those in the control group at all follow-up points.

These significant differences between the intervention groups and the control group in the use of MFPMs can be attributed to the limitations of the traditional counseling model in Jordan. Studies in Jordan identified different factors influencing women’s decisions and use of contraceptives in Jordan, including gender inequality and unequal power dynamics [25,26]. These studies reported that the counseling process in primary care is criticized by women for being inappropriate, very short, lacking privacy, ignoring women’s worries about using MFPMs, and not adequately informing women about different methods and their possible side effects [25,26,27].

The family planning decision-making process is complex, and it is impossible to guarantee the formulation of positive reproductive intentions or the translation of positive intentions into behavior without considering the irrationalities of human decision making. Current family planning programs and interventions in Jordan seem to rely on traditional economic theory, which assumes a falsely guaranteed rationality of human behavior and that people do exactly what is expected of them, are capable of processing large amounts of information, and make rational choices [12]. Behavioral economics, on the other hand, recognizes that human decisions are error-prone and offers several strategies to bridge the gap between favorable intentions and actual behaviors [12]. 

The study findings are in line with the recent adaptation of BE strategies in many health-related areas, including family planning and reproductive health [15]. Reminders such as text messages, framing, and identity priming were reported to be potentially effective BE strategies to address some of the cultural and information asymmetries that contribute to family planning decisions [15]. Positive behavior change outcomes were reported in 13 of the 14 studies examined in a meta-analysis of text messaging-based interventions [28]. Other studies reported that text-messaging intervention can lead to improvements in reproductive health knowledge and has the potential to reduce pregnancy risk among adolescent girls, with benefits potentially persisting after the intervention ends [29,30]. For family planning, a recent review of existing evidence on the use of BE strategies in family planning found that BE approaches can be used effectively to achieve a range of family planning outcomes, such as enhancing the use of modern contraceptives in Kenya using the combination of free contraception and an SMS reminder [23]. Researchers also found that BE strategies were effective in reducing injectable contraceptive discontinuation rates in Ethiopia through appointment cards used as reminders requiring return appointments and job-counseling assistance, which is a simple guide to frame the service provision interaction with clients [31]. 

BE-based interventions appear to be a promising method to improve family planning programs in Jordan for the benefit of both providers and users. Studies from Jordan have shown that midwives in the Ministry of Health provide inadequate services in government clinics [27], with the current study evidence suggesting that BE can improve midwives’ practices and service provision outcomes. It is recommended that counseling protocols be aligned with BE strategies, in terms of identity priming and framing strategies examined in the study and in terms of the design of manuals used by midwives for counseling women. It might be a good strategy to design the manuals to show and inform the clients about the most effective methods first and avoid any arbitrary designs. Policies that use reminders, such as text messages and phone calls, to remind people to use contraceptives or attend a family planning appointment can increase their use.

Other BE-based interventions include offering incentives or rewards for high levels of adherence to contraceptives or to midwives based on quality of care. At a higher policy level, introducing a standard package of the most effective contraceptives might be a promising approach in which women are automatically provided with the package unless they opt out. Policies that promote family planning as a normative behavior and policies that promote positive peer effects can help increase family planning use. Social marketing campaigns that promote family planning as a responsible behavior can help create a social norm that encourages its use.

One of the limitations of the study is the relatively small number of clinics involved; another is that the study was conducted in only two of Jordan’s governorates. It is possible that some differences between the centers might partially explain some of the differences in contraceptive continuation between the groups. However, our multivariate analysis attempted to adjust for some important characteristics that might be different between the study groups. Another limitation was that the required sample size for the control arm was not achieved, which could potentially impact the power of the study to detect differences between groups. Further studies covering the different governorates in Jordan and considering the subgroups of the population are recommended.

## 5. Conclusions

Simple BE-based interventions can be effective in enhancing women’s use of modern contraceptives. However, we believe that these findings merit consideration of replication of the project on a larger scale and over a somewhat longer time period to assess the longer-term impacts of BE interventions on fertility rates, maternal mortality, and other important outcomes.

## Figures and Tables

**Table 1:** The sociodemographic and clinical characteristics of women at the baseline.

Variable	Group						Total N = 1032	*p*-Value *
	Control (n = 295)		Counseling Only (n = 326)		Counseling and Messages (n = 411)			
	n	%	N	%	n	%	N	
**Age (year)**								
<25	80	27.1	109	33.4	146	35.5	335	0.01
25–30	113	38.3	140	42.9	150	36.5	403	
>30	102	34.6	77	23.6	115	28.0	294	
**Nationality**								<0.001
Jordanian	261	88.5	318	97.5	383	93.2	962	
Syrian	34	11.5	8	2.5	28	6.8	70	
**Mother’s education**								0.003
High school or less	146	49.5	200	61.3	207	50.4	553	
More than highschool	149	50.5	126	38.7	204	49.6	479	
**Husband’s education**								0.93
High school or less	204	69.2	221	67.8	282	68.6	707	
More than highschool	91	30.8	105	32.2	129	31.4	325	
**Occupation**								0.23
Housewife	235	79.7	272	83.4	347	84.4	854	
Employed	60	20.3	54	16.6	64	15.6	178	
**Income (JD)**								0.48
<400	120	41.0	137	43.5	157	39.1	414	
400+	173	59.0	178	56.5	245	60.9	596	
**Number of boys**								0.95
None	68	23.1	77	23.6	101	24.6	246	
1	123	41.7	130	39.9	158	38.4	411	
2	68	23.1	71	21.8	96	23.4	235	
>2	36	12.2	48	14.7	56	13.6	140	
Number of girls								0.33
None	75	25.4	105	32.2	100	24.3	280	
1	108	36.6	110	33.7	160	38.9	378	
2	58	19.7	61	18.7	80	19.5	199	
>2	54	18.3	50	15.3	71	17.3	175	0.22
**Smoking**	19	6.4	11	3.4	63	15.3	93	<0.001
**Anemia**	70	23.7	58	17.8	85	20.7	213	0.19
**Hypertension**	13	4.4	23	7.1	12	2.9	48	0.03
**Prediabetes**	2	0.7	4	1.2	2	0.5	8	0.51
**Gestational diabetes**	6	2.0	7	2.1	2	0.5	15	0.11
**Preeclampsia**	5	1.7	7	2.1	6	1.5	18	0.78

* *p*-values refer to the differences in women’s baseline characteristics between the study groups (between-group differences).

**Table 2:** Pregnancy-related and obstetric characteristics of women at the baseline.

Variable	Group						Total N = 1032	*p*-Value
	Control (n = 295)		Counseling Only (n = 326)		Counseling and Messages (n = 411)			
	n	%	n	%	n	%	N	
**Number of antenatal visits**								<0.001
0	40	13.6	90	27.6	19	4.6	149	
1–8	111	37.6	121	37.1	143	34.8	375	
>8	144	48.8	115	35.3	249	60.6	508	
**Time at first visit**								<0.001
First trimester	212	71.9	215	66.0	345	83.9	772	
Second trimester	23	7.8	16	4.9	22	5.4	61	
Third trimester	20	6.8	5	1.5	25	6.1	50	
No visit	40	13.6	90	27.6	19	4.6	149	
**Multiplicity**								0.90
Single	283	95.9	314	96.3	397	96.6	994	
Twin	12	4.1	12	3.7	14	3.4	38	
**History of stillbirth/neonatal mortality**	102	34.6	102	31.3	117	28.5	321	0.22
**Perception of the timing of the last pregnancy**								<0.001
Appropriate time	216	73.2	249	76.4	303	73.7	768	
Good but not the best time	59	20.0	20	6.1	71	17.3	150	
Wrong time	20	6.8	57	17.5	37	9.0	114	
**Place of delivery**								0.13
Hospital	292	99.0	316	96.9	405	98.5	1013	
Home	3	1.0	10	3.1	6	1.5	19	
**Type of delivery**								0.80
Vaginal	198	67.1	213	65.3	266	64.7	677	
Cesarean section	97	32.9	113	34.7	145	35.3	355	
**Gender**								0.48
Male	158	53.6	177	54.3	206	50.1	541	
Female	137	46.4	149	45.7	205	49.9	491	
**Birthweight**								0.10
Normal	261	88.5	303	92.9	365	88.8	929	
Low birthweight	34	11.5	23	7.1	46	11.2	103	
**Gestational age**								0.08
Full-term	272	92.2	308	94.5	395	96.1	975	
Premature	23	7.8	18	5.5	16	3.9	57	
**Intend to use family planning methods following latest delivery**	250	86.6	291	89.7	360	89.8	901	0.15

**Table 3:** Multivariate analysis of differences in modern family planning use between the study groups.

	3 Months				6 Months				9 Months			
	OR	95% CI		*p*-Value	OR	95% CI		*p*-Value	OR	95% CI		*p*-Value
**Group**												
Control	1											
Counseling only	1.6	1.1	2.3	<0.01	1.9	1.3	2.8	<0.001	2.0	1.4	3.0	<0.001
Counseling and reminder messages	1.7	1.2	2.4	<0.01	1.9	1.4	2.7	<0.001	2.3	1.6	3.2	<0.001
**Intention to use family planning (yes vs. no)**	2.0	1.3	3.3	<0.01	4.8	2.6	8.8	<0.001	4.4	2.4	8.1	<0.001
**Aware of family planning methods available at the clinic (yes vs. no)**					2.0	1.4	2.8	<0.001	1.7	1.2	2.4	<0.01
**Ever used any modern contraception (yes vs. no)**					1.9	1.4	2.5	<0.001	2.1	1.6	2.8	<0.001
**Place of delivery (home vs. hospital)**					6.5	1.8	24.0	<0.01	4.7	1.5	15.3	<0.01
**Multiplicity (single vs. twins)**					2.5	1.1	5.8	<0.03	2.3	1.0	5.3	0.05
**Reason for current visit (postpartum care vs. immunization)**	1.6	1.0	2.5	0.04								
**Husband’s education**												
High school or less	1.6	1.1	2.1	<0.01								
More than high school	1.0											
**Income (<JOD 400 vs. JOD 400+)**	1.4	1.0	1.8	0.04								
**Feeling about the timing of the last pregnancy**												
Appropriate time	1.0											
Good but not the best time	0.8	0.6	1.3	0.41								
Wrong time	2.0	1.3	3.2	<0.01								
**Heard about family planning (yes vs. no)**	1.4	1.0	2.0	0.03								
**Breastfeeding (yes vs. no)**	1.4	1.0	1.9	0.05								

## Data Availability

The data presented in this study are available from the corresponding author upon reasonable request.
